# Integrating disability and language access education in undergraduate medical training: a curriculum mapping study at a rural medical school

**DOI:** 10.1080/10872981.2026.2627020

**Published:** 2026-02-11

**Authors:** Lesley Cottrell, Scott Cottrell, Isabela Negrin, Anna Schuster, Megann Boone, Norman Ferrari

**Affiliations:** a Department of Pediatrics, School of Medicine & Center for Excellence in Disabilities, West Virginia University, Morgantown, WV, USA; b Department of Medical Education, School of Medicine, West Virginia University, Morgantown, WV, USA; c Department of Pediatrics, School of Medicine, West Virginia University, Morgantown, WV, USA; d School of Medicine, West Virginia University, Morgantown, WV, USA

**Keywords:** Medical education, cultural competence, disability care, competency development, English as a second language, patient care

## Abstract

This study offers a detailed analysis of how disability and English as a Second Language (ESL) competencies are incorporated into undergraduate medical curricula. Using systematic curriculum mapping at a rural LCME-accredited medical school, the research quantifies the hours dedicated to these areas—approximately 202 hours for disability-related topics and 177 hours for ESL—distributed across all four years. The curriculum emphasizes foundational knowledge in social determinants of health, cultural humility, communication strategies, and health disparities, with increasing clinical application throughout training. Early stages focus on core concepts, such as understanding disability definitions and culturally sensitive communication, which are reinforced during clinical rotations involving rural health, pediatrics, and internal medicine. The curriculum aims to develop skills gradually, culminating in leadership and advocacy roles by the final year. The analysis highlights the importance of longitudinal reinforcement and integration to ensure sustained competency development and effective application in clinical settings. Despite the comprehensive overview, the study’s limitations include its focus on a single institution and reliance on quantitative data, without assessing actual student competency or exploring extracurricular learning experiences. The findings underscore that a developmental, interconnected approach—combining knowledge, skills, and application—is essential to prepare physicians to deliver equitable, culturally competent care to diverse populations. Such curriculum design can help address health disparities, improve patient outcomes, and promote health equity. The study advocates for ongoing curriculum refinement that emphasizes continuous reinforcement and integration of disability and ESL-related competencies throughout medical training, ensuring future clinicians are equipped to meet the needs of increasingly diverse patient populations.

## Introduction

In an increasingly diverse healthcare environment, medical education must evolve to prepare future practitioners for the complexities of patient care that encompass various cultural, linguistic, and physical needs. The World Health Organisation [1] has highlighted that individuals with disabilities represent approximately 15% of the global population, yet medical curricula often inadequately address the specific health needs of these patients [2,3]. Similarly, the predominance of English as a second language (ESL) among patients poses additional challenges in communication, which is fundamental to effective medical care [4]. Despite the recognition of these issues, there remains a significant gap in the structured training provided to medical students in these critical areas [5].

These challenges are particularly pronounced in regions such as Appalachia, which spans parts of 13 states in the eastern United States. Communities in this region frequently face geographic isolation, limited access to healthcare [6,7], and a high prevalence of chronic health conditions. Socioeconomic factors, including lower income levels, higher rates of unemployment, and limited educational resources, further exacerbate health disparities [8,9]. Patients with disabilities in Appalachia may experience barriers related to mobility, communication, and health literacy, while those for whom English is a second language may encounter difficulties in understanding medical instructions or effectively communicating symptoms. These factors underscore the need for medical education to provide additional training that equips future practitioners to address the unique needs of Appalachian populations effectively.

Cultural considerations are also paramount. Appalachian communities often have strong local traditions, close-knit social networks, and varying levels of health literacy, which can influence health behaviours and trust in healthcare providers. Medical students and trainees must learn culturally sensitive communication techniques and ways to build rapport with patients who may be sceptical of the healthcare system or have differing health beliefs [10].

Moreover, rural Appalachian populations include patients with disabilities and those who speak English as a second language, who may face amplified barriers to care due to transportation limitations, lack of specialised services [11], and communication challenges. Training future providers in disability-inclusive practices, cross-cultural communication, and accessible care delivery is essential to improving health outcomes in these communities.

By integrating these region-specific considerations into medical education, institutions can better prepare future practitioners to provide equitable, effective, and contextually informed care. Exposure to rural healthcare settings during training also fosters adaptability, resourcefulness, and enhanced clinical decision-making skills—qualities essential for serving both Appalachian communities and other medically underserved populations.

The purpose of this manuscript is to catalogue the current undergraduate medical school curriculum related to patient care for individuals with disabilities and those who speak English as a second language, examining existing presentations, lectures, and other learning opportunities. These two patient groups were identified by a community advisory council for the Centre for Excellence in Disabilities as two groups of increasing representation in this region where additional training was desired. This analysis is informed by studies indicating that comprehensive training can significantly enhance student confidence and competence when interacting with diverse patient populations [2,12]. By systematically identifying and evaluating the resources currently employed in medical education, we aim to highlight effective pedagogical techniques and suggest enhancements to the curriculum that better align with the evolving needs of the communities we serve.

Furthermore, establishing a clinical environment designed for all patients not only benefits future healthcare providers but also improves health outcomes for marginalised groups [2]. The integration of accessible pedagogical strategies and culturally competent practices can facilitate the delivery of equitable care to all patients, bridging gaps that have historically hindered access for individuals with disabilities and ESL patients [13]. Through this examination, we hope to encourage medical educators to adopt and implement innovative learning interventions that promote understanding and empathy towards all patients, thereby enriching the training of medical practitioners who will be the frontline caregivers in our diverse society.

## Methods

To catalogue the learning events across the undergraduate medical school curriculum related to patient care for individuals with disabilities and those who speak English as a second language (ESL), we adopted a cross-sectional study design incorporating a single-institution curriculum mapping exercise. Our mixed-methods analyses involved several key steps, outlined as follows:

### Curriculum mapping

We initiated a comprehensive mapping of the undergraduate medical curriculum spanning from the first year (MS1) to the fourth year (MS4) within a rural allopathic medical school. As a Liaison Committee on Medical Education (LCME) accredited medical doctor (M.D.) degree programme, our educators must have a system to monitor content and ascertain how students acquire and demonstrate learning objectives across a competency-based framework. There are different models for curriculum mapping, which is essential for curriculum management and oversight. The medical school implements an internally developed web-based system. All course and clerkship directors, as well as individual learning event instructors, refer to a menu of “tags” or keywords to detail how specific content is linked to instructional methods and assessment methods [14]. Tags are assigned to all learning events to review offerings by theme and for curricular evaluation purposes outside of this study. For example, when educators populate disability content (e.g. lecture, simulation description, small-group case) into our online curriculum management system, one of the tags is labelled “disability.” Once tagged, all educators and students can search for these tags to identify a comprehensive list of learning events that target disability-related content. This system also allows a search for key words that are included into content, such as “disability” or language. This system is an important tool to identify and sequence content into an integrated and cohesive curriculum.

All key words and tags for events specifically focused on or relevant to the care of patients with disabilities and ESL patients were searched. This mapping process was conducted by a team of researchers familiar with medical education and inclusivity in healthcare.

### Measures

For each identified learning event, the following specifics were recorded: course/event title; date; delivery method; and topic focus area. We used a structured data-collection form to ensure consistency in capturing this information.

### Procedures

We analysed the published learning objectives of each course/event to ascertain their relevance to our two targeted areas of patient care. We conducted a series of consultation meetings with faculty experts in disability studies and language access in healthcare. These discussions helped to identify additional learning objectives deemed crucial for effective patient care that might not have been explicitly articulated in the original curriculum. Learning events were compared against both the stated intended objectives and the expert-identified important objectives. The research team employed a qualitative analysis framework to categorise and summarise findings, ensuring a thorough evaluation of the curriculum. The activities performed in this study were not deemed as human subjects research as it involved a review of education-based information already gathered.

### Analyses

We conducted descriptive statistics to calculate the frequencies and percentages for each identified learning event, detailing the distribution of topics covered across the MS1-MS4 timeline. A thematic analysis was performed on the qualitative data collected from the expert consultations and course/event summaries. This analysis aimed to identify common themes, gaps, and opportunities for enhancing teaching related to patient care for individuals with disabilities and ESL patients. The comparative data allowed us to identify gaps in the curriculum where essential topics and objectives were lacking. We categorised these gaps into themes to prioritise potential curricular improvements. Finally, our findings were synthesised into a comprehensive report outlining the structure of learning events, their alignment with established objectives, and the identified curricular gaps. This report aimed to serve as a foundation for proposed curricular improvements, emphasising strategies for integrating comprehensive and inclusive training into the medical education framework.

## Results

### Undergraduate medical curriculum overview

The undergraduate medical education curriculum at West Virginia University (WVU) School of Medicine is designed to prepare students for comprehensive clinical practice through a blend of foundational sciences and hands-on training. Located primarily in Morgantown, West Virginia, with clinical regional campuses in Charleston, and Martinsburg the WVU School of Medicine serves a diverse rural and underserved population, emphasising community engagement and primary care. The institution is characterised by its relatively small class sizes, typically enroling around 115 students per year, which fosters a close-knit learning environment that promotes mentorship and individualised attention. The curriculum encompasses approximately 4,000 hours of coursework, including lectures, clinical skills training, and experiential learning, integrating traditional lecture-based instruction with problem-based learning, early clinical exposure, and interprofessional education. This comprehensive approach aims to produce competent physicians equipped to address the unique healthcare needs of West Virginia and beyond.

### Disability competencies and hours

**Figure 1.**
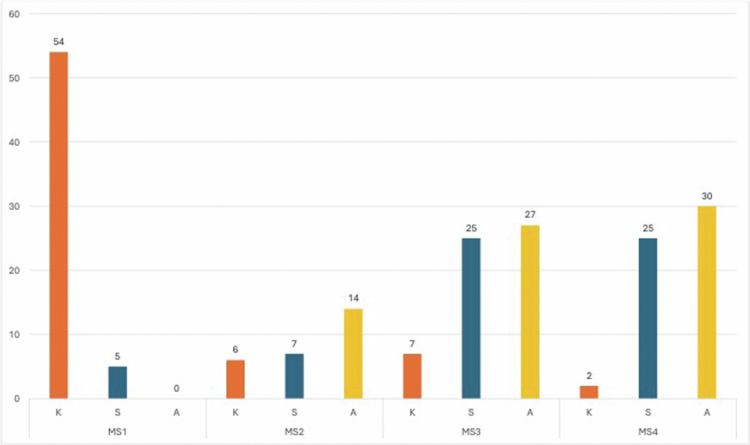
Medical student competencies and total training hours on disability and patient care: Note. Learning types included: Knowledge (K), Skills (S), and Application (A).

Our curriculum mapping search for tags and keywords identified 202 total baseline curriculum hours focused on disability and disability-related issues. Learners had additional potential to obtain more hours of training in the last two years of the curriculum within their clerkship and internship experiences. We have organised the specific learning events based on when they were introduced within the curriculum. Additionally, the type of outcome intended by the learning event (knowledge, skills, application) is listed in Figure 1.

### Medical school: first year (MS1)

Throughout the first year of medical school (MS1), students experience 55 learning events focused on conditions that are classified as disabilities, disability services, models, and approaches, and have the opportunity to practice these skills. At the beginning of medical school, students are introduced to a broad range of competencies across various disciplines. The CCMD 812 Physical Diagnosis and Clinical Integration (PDCI-2) course provides 3 hours dedicated to eliciting patients’ perspectives on health and illness, respecting communication differences, and developing therapeutic alliances—skills fundamental to patient-centred care. Here, students practice adapting their communication for patients with hearing loss (e.g. maintaining eye contact, using interpreters, minimising background noise) and vision impairments (e.g. providing verbal descriptions of procedures, offering accessible written materials). Similarly, CCMD 813 Neuroscience and Human Behaviour offers 2 hours focusing on team-based approaches to neurological conditions such as stroke and traumatic brain injury, where students are introduced to challenges in supporting patients with cognitive or speech impairments and working collaboratively with speech-language pathologists and occupational therapists.

Students also engage with public health concepts through CCMD 816, a one-hour lecture distinguishing impairment from disability, and a 1.5-hour overview of healthcare disparities in the United States (US) and West Virginia (WV), emphasising societal influences on health. The course CCMD 802 on Professional Development explores Appalachian and rural cultural impacts on health, with 3 hours highlighting the unique health beliefs and values prevalent among WV residents, including the significant veteran population that often faces service-related physical and psychological disabilities.

Problem-based learning (PBL) sessions across MS 1 and MS 2 (6 hours) emphasise real-world cases in which disability intersects with social determinants of health. For example, one case requires students to adapt discharge planning for a coal miner with respiratory disability and low health literacy, while another challenges them to coordinate resources for a child with developmental disabilities in a rural county lacking specialty service. Students also complete 2 hours on identifying risk factors for long-term disability, such as diabetes-related amputations, and 3 hours on ethical, legal, and sociological issues affecting disabled patients and practitioners.

Communication skill development is explicitly woven into these experiences. Across multiple sessions (ICS1 and ICS3, totalling 6 hours), students practice strategies such as slowing speech for patients with cognitive disabilities, offering multiple communication modalities for those with speech impairments, and ensuring informed consent through teach-back techniques for individuals with limited literacy. Faculty debriefings reinforce awareness of how communication needs differ across disability types, requiring preemptive strategies like using accessible formats, engaging interpreters, and modifying the clinical environment.

### Medical school: second year (MS2)

During the second year of medical school (MS2), students further develop disability-related competencies through a combination of rural health rotations and a total of 27 structure learning events focused on disability, chronic conditions, treatment planning, and patient-centred approaches to care. Building on foundational concepts introduced in MS1, these experiences emphasise clinical reasoning and real-world application within diverse practice settings. Students participate in disability-focused workshops and community-based placements that address barriers commonly encountered by patients with disabilities, including limited access to specialty care, transportation challenges, and gaps in rehabilitation services in rural and underserved areas.

MS2 learning events provide students with opportunities to apply clinical skills while engaging directly with patients and families, such as counselling caregivers of children with autism spectrum disorder, adapting care plans for patients with mobility limitations, and coordinating interdisciplinary supports across medical and community systems. Instruction and assessment extend beyond diagnostic accuracy to include communication skills, shared decision-making, and the ability to convey treatment plans clearly and respectfully to patients and caregivers with varied cultural, educational, and functional backgrounds. Through these longitudinal and applied experiences, students actively integrate disability-related knowledge and skills acquired in MS1 into clinical encounters, reinforcing competence, confidence, and professional responsibility in caring for patients with disabilities.

### Medical school: third year (MS3)

During the third year of medical school (MS3), students participate in 59 learning events focused on disability and patient care, reflecting a curricular shift from knowledge acquisition to immersive, practice-based learning. MS3 emphasises the integration of clinical reasoning with systems-based practice, professionalism, and ethical responsibility as students care for patients with disabilities across required clerkships. Disability-related learning during this year is embedded within authentic clinical environments, where students encounter the complex interplay of medical, social, and environmental factors that shape health outcomes.

Within the Internal Medicine Clerkship alone, which includes over 80 hours of instruction and supervised clinical care, students further develop competencies related to healthcare disparities and access, with particular attention to geographic and socioeconomic barriers affecting rural populations. Students learn to identify and address challenges such as limited transportation, reduced access to specialty and rehabilitation services, health literacy barriers, and fragmented social support systems, factors that disproportionately affect patients with disabilities. These themes are reinforced through clinical encounters and structured discussions addressing global disability and tropical medicine, which expand students’ understanding of disability beyond the local Appalachian context and highlight disability as a global public health concern.

Case-based seminars and clinical teaching sessions challenge students to apply disability-responsive clinical decision-making in diverse contexts, including chronic disease management, functional impairment, and comorbid conditions. Through these experiences, students are encouraged to consider disability not only as a medical condition but also as a lived experience shaped by social determinants of health, healthcare, infrastructure, and cultural context. This approach fosters systems-level thinking and prepared students to advocate for patients navigating complex care environments.

Patient-centred communication and ethical practice remain core competencies throughout MS3 and are increasingly emphasised as students assume greater clinical responsibility. Patient-centred communication is broadly defined as an approach in which healthcare providers actively engage with patients to understand their preferences, values, cultural context, and unique needs, and use this understanding to guide clinical decision-making [15]. Building on foundational instruction from the preclinical years, students apply patient-centred communication strategies during direct patient care, focusing on understanding patients’ values, preferences, and functional needs and incorporating these into shared decision-making. Clinical evaluations access students’ ability to communicate effectively with patients and caregivers with diverse backgrounds, including individuals with cognitive, sensory, or communication disabilities and those with limited English proficiency.

Across clerkships, students practice adapting communication to ensure informed consent, patient autonomy, and meaningful participation in care. Examples include modifying explanations for patients with cognitive disabilities, collaborating with interpreters during complex treatment discussions, and supporting families in paediatric or chronic care settings. Faculty observation and feedback during rounds, patient encounters, and debriefings reinforce inclusive communication strategies, ethical sensitivity, and respect for patient dignity.

Through these longitudinal and clinically grounded experiences, MS3 operationalizes disability competence as a developmental outcome rather than a discrete learning objective. Repeated exposure to patients with disabilities across multiple clinical settings enables students to integrate medical knowledge, communication skills, and systems-based awareness, ensuring they are prepared to deliver equitable, respectful, and effective care to patients with disabilities as they progress toward residency training.

### Medical school: fourth year (MS4)

During the fourth year of medical school (MS4), the curriculum focuses on preparing students for the transition to residency and for emerging leadership roles in clinical practice, with sustained emphasis on disability, responsive patient care and health equity. Building on competencies developed in earlier years, MS4 students are expected to demonstrate increasing independence in clinical decision-making, advocacy, and systems-based practice, particularly in underserved and rural healthcare settings.

In this year of preparation and transition, students received 57 learning experiences related to disability and patient care. Clinical experiences during MS4 include advanced inpatient and outpatient rotations which students manage complex cases involving chronic illness, disability, and intersecting social determinants of health. Students are tasked with synthesising medical, functional, and psychosocial information to develop comprehensive care plans that are tailored to patients’ abilities, preferences, and access to resources. These experiences require students to coordinate care across disciplines, facilitate transitions between care settings, and identify community and rehabilitation resource that support patients with disabilities beyond the clinical encounter.

A key focus of MS4 is the application of evidence-based strategies to promote health equity and improve quality and safety for patients with disabilities. Students engage in activities that emphasise patient advocacy, quality improvement, and population health, including evaluating institutional practices and policies that affect access to care, accommodation, and continuity of services. Through these experiences, students develop skills in analysing healthcare systems, understanding resource allocation, and recognising how policy-level decisions contribute to disparities experienced by individuals with disabilities.

Professionalism and ethical practice remain central throughout MS4, with increased expectations for accountability, leadership, and reflective practice. Students are assessed on their ability to communicate effectively across cultural, linguistic, and functional differences; to uphold patient autonomy and informed consent and to promote health literacy through clear, accessible communication. Faculty feedback and structured reflection encourage students to examine how sociocultural factors influence patient outcomes and to refine their professional identity and biases as physicians committed to equitable care. Students are tasked with developing management plans tailored to diverse populations, advocating for patient safety and quality improvement initiatives, and mobilising community resources. Additionally, students are trained to evaluate policies impacting healthcare access and disparities, developing leadership skills in advocating for systemic change.

**Figure 2.**
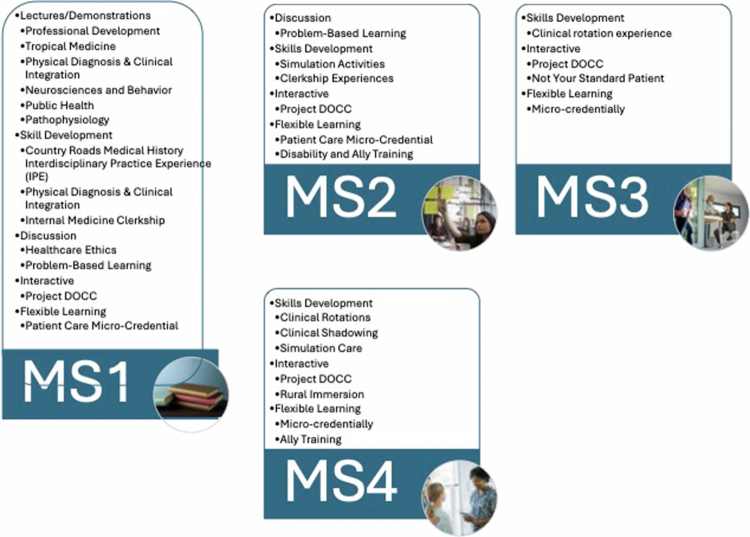
Overview of disability patient care concepts by training year and type.

Figure 2 illustrates the longitudinal integration of disability-related patient care concepts across the medical school curriculum from MS1 through MS4, highlighting the progressive complexity and application of learning experiences. In MS1, disability education is primarily delivered through foundational lectures, guided discussions, and introductory skill-development activities that establish core knowledge related to disability, health equity, communication, and social determinants of health. MS2 builds on this foundation through increased use of interactive learning experiences, applied skill development, and micro-credentialed training modules that emphasise clinical application, patient counselling, and community-based care, particularly in rural and underserved settings. During MS3, disability-related learning is embedded within clinical clerkships and case-based discussions, where students apply patient-centred communication, ethical reasoning, and systems-based practice in real-world patient care settings. In MS4, experiences emphasise advanced clinical responsibility, leadership, and advocacy, incorporating advanced clinical rotations, quality improvement initiatives, and policy-informed discussions that prepare students for residency and independent practice. Collectively, the figure demonstrates a developmental approach in which lectures and discussion evolve into interactive, skills-based, and credentialed clinical experiences, reinforcing disability competency as a core outcome of medical training.

### ESL competencies and hours

Across the four years of undergraduate medical education, learners receive approximately 177 total hours of curricular exposure related to patient care for individuals who speak limited English or for whom English is a second language (ESL). Instruction is intentionally sequenced to support progressive competency development, beginning with foundational communication knowledge in MS1 (approximately 40 hours), advancing through structured skills practice in MS2, and culminating in applied, real-world clinical experiences and leadership expectations during MS3 and MS4. Similar to disability-related competencies, ESL-related learning is reinforced longitudinally, with increasing emphasis on application during clinical clerkships and sub-internships.

### First year of medical school (MS1)

During MS1, students are introduced to foundational competencies required for effective communication with ESL patients, with an emphasis on awareness, knowledge acquisition, and early skill development. In CCMD 812 Physical Diagnosis and Clinical Integration (PDCI-2), approximately 3 hours are dedicated to structured instruction and practice in core communication skills, including eliciting patient perspectives on health and illness while attending to cultural explanations of symptoms and treatment; recognising and respecting verbal and nonverbal communication differences (e.g. tone, silence, body language, and eye contact); appropriate and ethical use of medical interpreting services, including addressing the patient directly, pacing information delivery, and confirming understanding; and establishing therapeutic alliances that reflect patients’ cultural values and linguistic preferences.

CCMD 813 Neuroscience and Human Behaviour extends these competencies by situating communication within team-based and neurologic care contexts, highlighting how cognitive, developmental, or language barriers affect patients and families. Students examine ethical and sociocultural considerations, such as culturally mediated interpretations of neurodevelopmental diagnoses and family decision-making roles.

In CCMD 814 Healthcare Ethics, a 3-hour module focuses on integrating linguistic and cultural factors into comprehensive history taking and treatment planning. Students practice developing evidence-based, culturally responsive care plans while avoiding assumptions about patient preferences. Instruction emphasises cultural humility strategies, including the use of open-ended questioning, avoidance of culturally loaded jargon, and respectful negotiation of care when traditional health practices are present.

Additional coursework, including CCMD 816 Public Health and CCMD 712, reinforces population-level perspectives on language, culture, and health, with particular attention to Appalachian communities. Students analyse how culture and geography shape health behaviours, identify contributors to medical error in cross-cultural encounters, and apply evidence-based approaches to reducing health disparities in rural and linguistically diverse populations.

### Second year of medical school (MS2)

In MS2, ESL competencies shift form primarily didactic instruction to structured skills development through experiential and simulation-based learning (approximately 19 hours). Programmes such as Project DOCC (Delivery of Chronic Care) and *Not Your Standard Patient* integrate self-advocate leaders and simulated patient encounters into the curriculum, allowing students to apply communication strategies in controlled settings.

Through role-played scenarios, students practice complex medical procedures to ESL patients with limited health literacy, navigating culturally sensitive topics (e.g. reproductive health), and responding to nonverbal indicators of misunderstanding. Emphasis is placed on anticipatory communication strategies, such as teach back methods, plain-language explanations, use of visual aids, and provision of translated or linguistically appropriate written materials. These experiences reinforce confidence and competence prior to entry into full clinical immersion.

### Third year of medical school (MS3)

**Figure 3.**
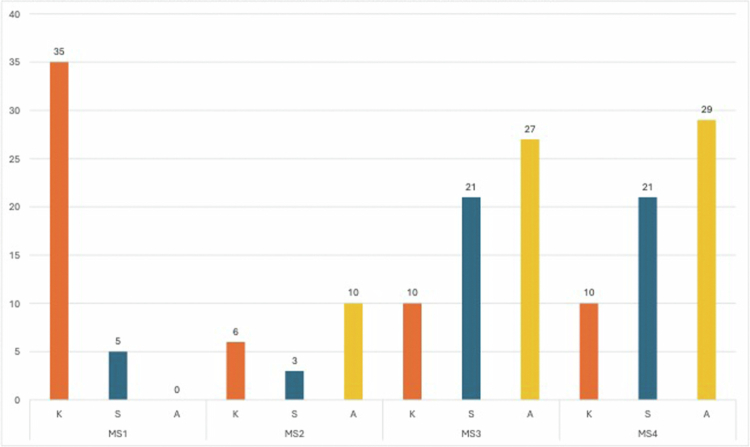
Medical student competencies and total training hours on ESL and patient care. Note: Learning types included: Knowledge (K), Skills (S), and Application (A).

During MS3, ELS-related learning is embedded primarily within required clinical clerkships, with approximately 58 hours of applied training documented (Figure 3). In the Internal Medicine Clerkship (80+ clinical hours with integrated ESL content), students encounter patients facing geographic, linguistic, and health literacy barriers common in rural settings. Learners apply communication strategies in real patient care, including simplifying medication instructions, using culturally relevant metaphors, and appropriately engaging family members in decision-making. Case-based discussions highlight system-level barriers to interpreter access, particularly in rural hospitals, and introduce advocacy strategies for promoting equitable communication resources within healthcare systems.

In OB-GYN and Paediatric clerkships, students receive targeted instruction and feedback on managing linguistically complex and culturally sensitive conversations, such as discussions of contraception, prenatal care, childhood development, and developmental delays. Skills emphasised include pacing difficult conversations, ensuring informed consent across language barriers, and collaborating effectively with interpreters when terminology does not directly translate across languages.

### Fourth year of medical school (MS4)

In MS4, ELS competencies are assessed at the level of professional practice and leadership, with approximately 60 hours of advanced application documented across sub-internships and clinical electives (see Figure 3). During hospital-based sub-internships, students are evaluated on their ability to: communicate effectively across linguistic, cultural, and socioeconomic differences; adapt to diverse values and expectations regarding healthcare decision-making; and independently employ advanced strategies, such as interpreter pre-briefing, recognition of nonverbal comprehension cues, and co-creation of culturally acceptable care plans with patients and families. At this stage, learners are expected to function as advocates and emerging leaders, identifying institutional and systemic barriers (e.g. limited interpreter availability) and proposing workflow or policy improvements to enhance language access and equity.

## Discussion

This study highlights the significant emphasis placed on disability and ESL competencies within the undergraduate medical curriculum, providing a detailed overview of the hours dedicated to these areas and how they are integrated throughout the training years. Importantly, examining what medical students receive in these domains is vital because it directly influences their preparedness to deliver equitable, culturally sensitive, and patient-centred care to diverse populations. As healthcare increasingly recognises the importance of addressing social determinants, cultural competence, and disability, ensuring that medical education effectively teaches these competencies is crucial for reducing disparities and improving health outcomes [16,17].

Our findings demonstrate that the curriculum builds a strong foundational knowledge base early in training, including core concepts such as understanding disability definitions, social determinants of health, and communication strategies with diverse patient populations. This foundational knowledge is essential, as it provides the scaffolding upon which more complex skills and application-based experiences are later developed. For example, initial coursework introduces learners to key concepts, which are then reinforced and expanded during clinical rotations, where they encounter real-world challenges related to disability, language barriers, and health disparities. This developmental sequence, beginning with knowledge acquisition and advancing through skills development to applied practice, is consistent with established educational frameworks that emphasise longitudinal competency development in medical training [18–20].

However, an important consideration is the sustainability and reinforcement of these competencies throughout the later years of training. While early curriculum components establish necessary knowledge and skills, maintaining and deepening this understanding during clinical years is vital for connecting all concepts into a cohesive, integrated approach to patient care. Without ongoing reinforcement, initial knowledge about cultural humility or health disparities may diminish, reducing the likelihood that students will consistently apply these principles in practice [21,22]. Therefore, curriculum designers should consider strategies for longitudinal reinforcement—such as repeated exposure, reflective practice, and integrated assessments—to sustain and deepen competencies in disability and ESL-related areas throughout the entire training continuum.

Moreover, fostering the integration of knowledge, skills, and application is essential for cultivating physicians capable of addressing complex, multifaceted health issues faced by vulnerable populations. Future curriculum development should prioritise opportunities for students to synthesise concepts—connecting their understanding of social determinants, communication strategies, ethical considerations, and health systems—within clinical contexts [23,24]. Such integration not only enhances learner competence but also prepares future physicians to deliver holistic, patient-centred care that addresses both medical and social needs. This approach aligns with the competency-based medical education paradigm that emphasises the importance of bridging theory with practice [25].

This study has several limitations, notably that it is based on curricular mapping data from a single allopathic medical school in a rural setting, limiting its generalisability to other institutions with different curricula or geographic contexts. It primarily provides quantitative information on hours dedicated to disability and ESL competencies without assessing the quality or effectiveness of instruction or the actual competency development of students, Additionally, informal or extracurricular learning experiences that may contribute to skill acquisition were not captured, and the findings represent a snapshot in time, which may not reflect ongoing or future curriculum changes aimed at enhancing these areas. Future research should include assessments of learner outcomes, longitudinal evaluations, and broader institutional samples to better understand the impact of curriculum design on competency development and patient care.

## Conclusion

In conclusion, our findings underscore the importance of a comprehensive, developmental approach to teaching disability and ESL competencies, emphasising both initial knowledge acquisition and the sustained, integrated application of these skills throughout medical training. Ensuring ongoing reinforcement and connection across the curriculum will be critical for producing clinicians equipped to meet the diverse needs of the populations they serve, ultimately advancing health equity and quality of care [26].

## Data Availability

Data for this study are available upon request to the corresponding author.
